# Regulatory Mechanisms of Microbial Consortium Inoculant SynCom-SASW01 in Modulating Rhizosphere–Endophytic Interactions and Enhancing Drought Resistance in Wheat

**DOI:** 10.3390/microorganisms14071396

**Published:** 2026-06-24

**Authors:** Chaofeng Yu, Mengjie Zhang, Wenya Xing, Xin Dong, Rui Li, Yi Qu, Shuye Chen, Fangfang Xu, Fuying Feng, Jianyu Meng

**Affiliations:** College of Life Science, Inner Mongolia Agricultural University, Hohhot 010011, China; 15036761470@163.com (C.Y.); zmj_1127@163.com (M.Z.); 16639761243@sina.cn (W.X.); dongxin090526@163.com (X.D.); 18748129064@163.com (R.L.); m18975039003@163.com (S.C.); xufangfang2024@163.com (F.X.); foyefeng@hotmail.com (F.F.)

**Keywords:** branched-chain amino acid (BCAA) biosynthesis pathway, drought stress, rhizosphere microecological reshaping, synthetic microbial community, *Triticum aestivum* L.

## Abstract

Driven by increasingly severe drought stress associated with global warming, this study investigated a synthetic microbial community, SynCom-SASW01, with strong stress tolerance and plant growth-promoting potential, and systematically elucidated its mechanisms for enhancing drought resistance in wheat (*Triticum aestivum* L.). Dual-site field trials demonstrated that SynCom-SASW01 significantly alleviated drought-induced growth suppression, increasing grain yields by 10.42% and 8.52% at the Hohhot and Hulunbuir sites, respectively. This improvement was primarily associated with increased effective tiller number and enhanced root vigor. Physiologically, inoculation promoted root proline and glutathione accumulation and enhanced antioxidant enzyme activities, including superoxide dismutase, thereby reducing malondialdehyde levels. Environmental analyses showed that the consortium established rhizosphere “micro-reservoirs” through exopolysaccharide secretion, improving soil relative water content and the availability of alkali-hydrolyzable nitrogen and phosphorus. High-throughput sequencing revealed that SynCom-SASW01 reshaped the endosphere microbiome through early colonization priority effects, selectively enriching beneficial taxa such as *Pseudomonas*. Functional prediction indicated upregulated branched-chain amino acid biosynthesis, promoting osmotic adjustment and redox homeostasis. These findings provide a microbiome-based strategy for stabilizing wheat productivity in arid regions.

## 1. Introduction

The Sixth Assessment Report of the Intergovernmental Panel on Climate Change (IPCC) highlighted that global warming was intensifying surface evapotranspiration, which led to a significant increase in the frequency and severity of drought events. This trend further exacerbated global water scarcity and its impact on agricultural production [1]. Wheat (*Triticum aestivum* L.) is one of the world’s most vital dryland crops, with a global cultivation area of approximately 175 million hectares, and provides nearly 20% of total human caloric intake. However, wheat frequently encounters periodic or prolonged drought stress during its growth stages, posing a severe threat to global food security [2]. Although wheat has evolved various adaptive mechanisms, including morphological adjustments (e.g., optimized root-to-shoot ratios), osmotic regulation, and activation of antioxidant enzyme systems, these defenses can be overwhelmed. When stress intensity exceeds physiological thresholds, it induces metabolic disorders, accumulation of reactive oxygen species (ROS), and premature senescence, ultimately resulting in significant yield losses [3,4]. Given the limitations of intrinsic plant defense mechanisms under severe drought stress, increasing attention has been directed toward the role of plant-associated microorganisms in enhancing crop resilience.

In recent years, the rhizosphere microbial community has been increasingly recognized as the “second genome” of plants, playing a pivotal role in stress resistance [5]. Through complex plant–microbe interactions, rhizosphere microorganisms enhanced host drought tolerance by constructing a multi-layered defense system [6,7]. Drought stress can induce directional reshaping of the rhizosphere microbiome, enriching plant growth-promoting microorganisms (PGPMs) that improves host resilience by modulating metabolism, enhancing nutrient availability, and regulating phytohormone balance [8,9]. For instance, enrichment of Actinobacteria in the sorghum rhizosphere under drought conditions has been shown to significantly improve nutrient uptake and drought tolerance [10,11,12]. However, this natural “cry-for-help” mechanism exhibits a time lag, limiting its effectiveness during early seedling stages or under sudden, severe stress [13]. Therefore, artificial inoculation with exogenous microbial agents to modulate rhizosphere community structure at early growth stages has been considered an effective strategy [14,15]. Compared with traditional single-strain inoculants, which exhibit limited functionality and poor environmental adaptability, synthetic communities (SynComs), designed based on functional redundancy and ecological stability, offer significant advantages [16]. By mimicking the metabolic diversity of natural microbiota, SynComs enhance colonization success and functional stability through interspecies synergy, and represent a frontier in agricultural microbiology. Although the beneficial effects of SynComs have been increasingly reported, the underlying mechanisms through which synthetic microbial consortia reshaped the rhizosphere–endosphere microenvironment and enhanced drought resistance remain insufficiently understood.

Microbial-mediated drought resistance fundamentally depends on reshaping the “rhizosphere–endosphere” microecological continuum [17]. Microorganisms could directly regulate plant physiology by producing 1-aminocyclopropane-1-carboxylate deaminase, exopolysaccharides (EPS), and phytohormones such as indole-3-acetic acid [18]. Additionally, they could promote the formation of beneficial microecological barriers by competing for ecological niches and modifying root exudate compositions [19]. Based on extensive microbial resource mining and functional validation, the Laboratory of Environmental Microbiology and Biotechnology in Arid and Cold Regions at Inner Mongolia Agricultural University constructed a synthetic microbial community, designated SynCom-SASW01 [20]. Following the principles of functional complementarity and ecological compatibility, this synthetic consortium consists of five core strains, namely *Enterobacter hormaechei* FN0603, *Enterobacter cloacae* FWP0601, *Enterobacter ludwigii* FWP1205, *Pseudomonas putida* FWP0405, and *Stenotrophomonas maltophilia* HLPD6. The SynCom-SASW01 was further formulated with biochar and other additives to develop a stress-tolerant plant growth-promoting (PGP) inoculant. Preliminary studies indicated that this SynCom-SASW01 integrated multiple PGP and stress-resistance traits, including the production of IAA, ACC deaminase, and EPS, as well as efficient phosphate solubilization and siderophore production. Consequently, it exhibited strong potential to promote wheat growth and enhance resilience under saline-alkali stress. However, the mechanisms by which SynCom-SASW01 mediates material and signal exchange with the host under drought conditions remained unclear. Specifically, how it coordinately regulates antioxidant enzyme activities within the “rhizosphere–endosphere” system remains poorly understood. The pathways through which it modulates wheat physiological metabolism and reshapes microecological community structure during colonization require further investigation. These unresolved questions highlighted the need to investigate how SynCom-SASW01 interacts with wheat and its associated microbiome under drought conditions and how these interactions contribute to enhanced drought tolerance.

Based on these considerations, we hypothesized that SynCom-SASW01, through efficient early colonization, reshapes the microecological patterns of the wheat “rhizosphere–endosphere” system and overcomes the time-lag effect of host defenses. Through a cascade involving microecological succession, cross-kingdom signaling, and enhanced phenotypic defense, SynCom-SASW01 activates the wheat antioxidant defense system, thereby improving drought tolerance, promoting growth, and increasing yield. This study aimed to construct a multidimensional interaction model linking the exogenous consortium, rhizosphere/endosphere microenvironment, and host physiological metabolism. These findings provide experimental evidence for plant–microbe interaction mechanisms under stress and offer theoretical support for developing microbiome-based bioaugmentation strategies in arid agriculture.

## 2. Materials and Methods

### 2.1. Strain Activation and Identification

Five target bacterial strains (*E. hormaechei* FN0603, *E. cloacae* FWP0601, *E. ludwigii* FWP1205, *P. putida* FWP0405, and *S. maltophilia* HLPD6) were retrieved from ultra-low-temperature storage at −80 °C and gradually thawed to 4 °C in an ice box (strain isolation sites are listed in Supplementary Table S1, and the PGP characteristics of strains are presented in Supplementary Table S2). A 10 μL aliquot of each bacterial suspension was inoculated onto R2A agar medium (20 g·L^−1^, pH 7.0) and incubated at 28 °C for 24 h until visible colonies formed. Single colonies were selected for genomic DNA extraction using a resin-based extraction method. Species identity was confirmed by 16S rRNA gene sequencing targeting the V5-V7 regions on the Illumina MiSeq platform, followed by comparison with sequences in the NCBI GenBank database [21]. Verified strains were resuspended in sterile phosphate-buffered saline (PBS; pH 7.4). The optical density at 600 nm (OD_600_) was measured using a spectrophotometer(Shanghai INESA Scientific Instrument Co., Ltd., Shanghai, China) and adjusted to 1.0 ± 0.1, corresponding to approximately 10^8^ CFU·mL^−1^, by dilution or concentration as needed. Finally, the standardized bacterial suspensions were mixed at an equal 1:1:1:1:1 (*v*/*v*) ratio and vortexed thoroughly to assemble the synthetic community, SynCom-SASW01.

### 2.2. Microbial Inoculant Seed Coating

A homogeneous coating matrix was prepared by mixing the adsorbent (biochar), binder (maltodextrin), biopriming agent (trehalose), and additives (including phosphorus and potassium mineral powder, molasses, fulvic acid, and potassium silicate) with the SynCom-SASW01 suspension at a mass ratio of 40–50:10–15:8–10:4–5:1 (all experimental reagents and instruments are listed in Supplementary Tables S3 and S4) [20]. The formulation ensured a microbial load of ≥10^7^ CFU·g^−1^, with biochar (specific surface area: 800–1000 m^2^·g^−1^) serving as a controlled-release carrier. The coating matrix was applied to wheat seeds at a 1:20 (*w*/*w*) mass ratio. The mixture was thoroughly blended using a V-type mixer (15 r·min^−1^ for 10 min) and subsequently dried in the dark. Four experimental groups were established: NC (negative control, no treatment), CC (chemical coating only, without SynCom-SASW01), BC (seed-soaking with SynCom-SASW01 only), and BCC (biological coating consisting of SynCom-SASW01 and the chemical coating matrix).

### 2.3. Pot Experiment Design Under Drought Stress

The base soil was collected from a wheat field in Hohhot, Inner Mongolia, China (41°11′55″ N, 111°39′52″ E), air-dried, and passed through a 2 mm sieve. The cultivation substrate consisted of sand, selected base soil, and a trace amount of multi-source inoculum soil at a ratio of 1:1:0.01 (*w*/*w*/*w*). River sand and base soil were treated with three cycles of moist heat sterilization at 121 °C for 20 min each. After each sterilization cycle, the substrate was thoroughly mixed, cooled to room temperature, and then blended with a trace amount of multi-source inoculum soil. The substrate properties were as follows: total nitrogen, 1.406 g·kg^−1^; total phosphorus, 2.514 g·kg^−1^; total potassium, 18.296 g·kg^−1^; and organic matter, 33.258 g·kg^−1^. Each cylindrical pot (20 cm in height and 9 cm in diameter) was filled with 1 kg of substrate.

Moisture was adjusted using Hoagland nutrient solution (pH 7.0) to maintain 50–60% relative water content (RWC), representing moderate drought stress. Water loss was compensated daily based on gravimetric measurements. Wheat was sown at a density of 25 seeds per pot and grown to the three-leaf stage. Growth parameters, including plant height, stem diameter, shoot and root dry weight, and chlorophyll content, were recorded. Cultivation was conducted in a controlled greenhouse at 22 °C, with a light intensity of 13,000 lx and a 12 h light/12 h dark photoperiod.

### 2.4. Field Experiment Design

Comparative field trials were conducted at typical arid experimental sites in Hohhot (41°11′55″ N, 111°39′52″ E) and Hulunbuir (50°11′54″ N, 120°02′02″ E), Inner Mongolia. Two treatments were established: NC (control) and BCC (SynCom-SASW01 biological coating). Each treatment was implemented in standardized plots of approximately 0.34 hm^2^, using the drought-tolerant wheat variety LM35 sown by mechanized precision seeding at a density of 150 kg·hm^−2^.

Enhanced diammonium phosphate (N-P_2_O_5_-K_2_O ≥ 34-14-12), containing polypeptide-chelated potassium (K ≥ 12%) and zinc–boron (Zn + B ≥ 3%), was applied as basal fertilizer at 375 kg·hm^−2^ via furrow application. All other field management followed regional agronomic standards to ensure consistency in soil properties, operational timing, and local climatic adaptation.

### 2.5. Parameter Measurements

A systematic approach using multiple standardized indicators was employed to evaluate physiological responses and soil micro-environmental changes. Soil enzymes and plant stress-related indices were quantified using commercial assay kits (Grace Biotechnology, Suzhou, China), including peroxidase (POD, μmol·min^−1^·g^−1^), catalase (CAT, μmol·min^−1^·g^−1^), superoxide dismutase (SOD, U·g^−1^), malondialdehyde (MDA, nmol·g^−1^), proline (Pro, μg·g^−1^), reduced glutathione (GSH, μmol·g^−1^), and root vitality (Determined by the 2,3,5-triphenyltetrazolium chloride (TTC) staining method, μg·h^−1^·g^−1^). Soil physical properties and plant phenotypes were measured using standardized instruments: RWC (%), electrical conductivity (EC, μS·cm^−1^), and pH (1:2.5 soil-to-water ratio). Plant indicators included plant height (mm), stem diameter (mm), shoot/root dry weight (mg), penultimate leaf area (mm^2^), and relative chlorophyll content (SPAD value). Primary equipment included a chlorophyll meter (SPAD-502Plus, Konica Minolta, Tokyo, Japan), a multi-parameter analyzer (DZS-706-B, Lei-ci, Shanghai, China), and an analytical balance (FA2004B, Jinghai, Shanghai, China). Soil nutrients were determined by standard chemical methods: hydrolyzable nitrogen (HN, alkaline diffusion method), available phosphorus (AP, molybdenum-antimony colorimetric method), available potassium (AK, ICP-OES), and easily oxidizable organic carbon (EOC, potassium dichromate oxidation).

The biological permanent wilting coefficient was determined by withholding irrigation from a separate set of potted plants until permanent wilting symptoms was observed. Soil samples were then rapidly collected, and moisture content was measured determined using the oven-drying method [22].

Seedling quality was evaluated using the Dickson Quality Index (DQI) [23]:

$$DQI=\left(\frac{Stem\;Diameter}{Plant\;Height}+\frac{Root\;Dry\;Weight}{Shoot\;Dry\;Weight}\right)\cdot (Total\;Weight)$$

### 2.6. High-Throughput Sequencing and Data Analysis

High-throughput sequencing was performed by Personal Biotechnology Co., Ltd. (Shanghai, China), encompassing quality control, library construction, sequencing, and standard bioinformatics analysis. Raw FASTQ data and quality reports were provided. Sequence denoising was performed using the QIIME2 DADA2 (2024.5) pipeline to generate the amplicon sequence variant (ASV) table and representative sequences [24]. Taxonomic assignment was conducted using the QIIME2 Naive Bayes classifier based on the SILVA 138.1 database. Alpha rarefaction was performed using the qiime diversity alpha-rarefaction command (parameters: --p-steps 10 --p-min-depth 10 --p-iterations 10). The maximum rarefaction depth was set to 95% of the sequence count of the sample with the lowest depth. Alpha diversity indices were calculated as the mean value at the maximum rarefaction depth. Data were visualized as boxplots using R scripts (4.4.2), with significance verified via the Kruskal–Wallis test and Dunn’s post hoc test. Beta diversity was assessed using the qiime diversity core-metrics-phylogenetic or core-metrics commands to calculate Jaccard, Bray–Curtis, unweighted UniFrac, and weighted UniFrac distance matrices. Principal Coordinate Analysis (PCoA) was performed and visualized using QIIME2 View and R-based scatter plots. Functional potential prediction was conducted using PICRUSt2 (2.6.0) based on MetaCyc Level 3 pathways (https://metacyc.org/).

## 3. Results

### 3.1. Regulatory Effects of SynCom-SASW01 on Wheat Growth Phenotypes and Yield Under Drought Stress

**Figure 1:**
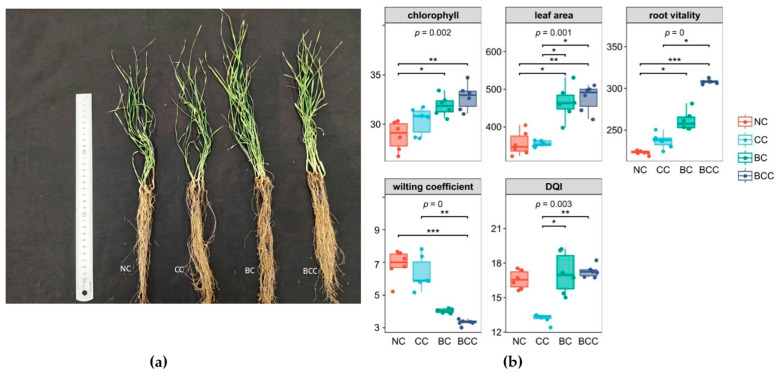
Effects of SynCom-SASW01 on wheat growth under drought stress. (**a**) Growth phenotypes of wheat under drought stress. (**b**) Effect of SynCom-SASW01 on key wheat growth indicators under drought stress. NC: control group; CC: chemical coating group (without SynCom-SASW01); BC: seed-soaking treatment with SynCom-SASW01 alone; BCC: biological coating treatment combining the SynCom-SASW01 consortium with a chemical coating matrix; chlorophyll: chlorophyll content of the penultimate leaf; leaf area: leaf area of the penultimate leaf; root vitality: root activity (determined by the TTC method); wilting coefficient: biological permanent wilting coefficient; DQI: Dickson Quality Index. Asterisks indicate significant differences: * *p* < 0.05, ** *p* < 0.01, and *** *p* < 0.001.

Phenotypic observations revealed that inoculation with the SynCom-SASW01 consortium effectively alleviated the inhibitory effects of drought stress on wheat growth (Figure 1a). Compared with the uninoculated control (NC), the inoculated treatment group (BCC) exhibited significant growth advantages under drought conditions, primarily attributable to improved plant water-use status. Specifically, the wilting coefficient of the BCC group was significantly reduced, while root vitality was significantly enhanced, indicating that SynCom-SASW01 improved the water acquisition and retention capacity of wheat under water-deficient conditions. In addition, the DQI, an indicator of overall plant vigor, was significantly increased following inoculation, further demonstrating the synergistic role of the inoculant in enhancing drought adaptability.

As photosynthesis provides the physiological basis for crop yield formation, this study further focused on the growth performance of the penultimate leaf (flag 1a). SynCom-SASW01 treatment significantly increased both leaf area and chlorophyll content of the flag-1 leaf (Figure 1b, *p* < 0.05). The enlarged leaf area and elevated photosynthetic pigment content collectively enhanced photosynthetic potential, thereby supporting carbon assimilation under drought stress. Statistical analysis showed that the BCC group exhibited significant improvements across all six key physiological indicators, suggesting that the inoculant systematically promoted host phenotypic remodeling.

To evaluate the practical application potential of SynCom-SASW01, dual-site field trials were conducted in Hohhot (a semi-arid region) and Hulunbuir (an alpine semi-arid region). The consistency of the results across geographically distinct environments demonstrated the strong ecological adaptability of the inoculant. At the Hohhot site, the BCC treatment increased grain yield by 10.42% compared with the NC treatment (3940.38 kg·ha^−1^ vs. 3568.65 kg·ha^−1^). Similarly, at the Hulunbuir site, yield increased by 8.52% (5021.58 kg·ha^−1^ vs. 4627.50 kg·ha^−1^). Further analysis of yield components revealed that the increase in effective tiller number was the primary driver of yield improvement (Table 1).

### 3.2. Impact of SynCom-SASW01 on the Antioxidant Defense System of the “Root–Soil” Continuum

**Figure 2:**
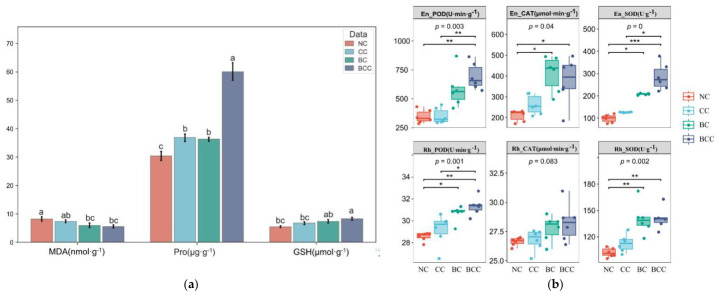
Regulatory effects of SynCom-SASW01 on osmolyte accumulation and antioxidant enzyme activities in the wheat root–soil system. (**a**) Osmolyte levels in root tissues; (**b**) Antioxidant enzyme activities in the root–soil continuum. NC: control group; CC: chemical coating group (without SynCom-SASW01); BC: seed-soaking treatment with SynCom-SASW01 alone; BCC: biological coating treatment combining the SynCom-SASW01 consortium with a chemical coating matrix. MDA: malondialdehyde; Pro: proline; GSH: glutathione; POD: peroxidase; CAT: catalase; SOD: superoxide dismutase. En: endosphere enzyme activity; Rh: rhizosphere soil enzyme activity. Different letters indicate significant differences between groups; Asterisks indicate significant differences: * *p* < 0.05, ** *p* < 0.01, *** *p* < 0.001.

As the primary organ for sensing drought signals, the root system plays a critical role in osmotic adjustment and redox homeostasis, both of which are key indicators of drought tolerance. Inoculation with SynCom-SASW01 significantly promoted the accumulation of osmolytes in wheat roots (Figure 2a). Specifically, the Pro content in the BCC treatment group was approximately two-fold higher than that in the control (NC) group, while the GSH content also increased significantly (*p* < 0.05). Concurrently, MDA content, an indicator of membrane lipid peroxidation, was lowest in the BCC group. These results suggest that SynCom-SASW01 effectively maintains root cell membranes integrity under drought stress by enhancing osmoprotective capacity.

Further analysis of antioxidant enzyme activities in the endosphere (En) (Figure 2b) revealed that SynCom-SASW01 exerted a potent immuno-inductive effect. Under BCC treatment, the activities of endospheric SOD (En_SOD), endospheric POD (En_POD), and endospheric CAT (En_CAT) were all significantly upregulated compared to the NC group, with the most profound induction observed in En_SOD (*p* < 0.001). This enhancement of endogenous antioxidant capacity establishes an efficient biochemical barrier for scavenging ROS, thereby strengthening antioxidant resilience at the tissue level.

Moreover, the stress defense response extended beyond plant tissues to the rhizosphere interface. SynCom-SASW01 inoculation significantly altered biochemical activity in the rhizosphere (Rh) microenvironment (Figure 2b). Compared with the NC group, BCC treatment significantly increased rhizosphere POD (*p* < 0.01) and SOD (*p* < 0.01) activities. Although the increase in CAT activity was not statistically significance (*p* = 0.083), an overall upward trend was observed following inoculation. The coordinated enhancement of enzymatic activities in both the endosphere and rhizosphere suggests that SynCom-SASW01 establishes a strong functional interaction with the host during colonization. In addition to directly contributing to the soil enzyme pool, SynCom-SASW01 may indirectly influence rhizosphere microecology functions by modulating root exudation patterns, thereby collectively enhancing wheat drought resistance and yield stability.

### 3.3. Impact of SynCom-SASW01 on Rhizosphere Environmental Factors and Soil Nutrients

**Figure 3:**
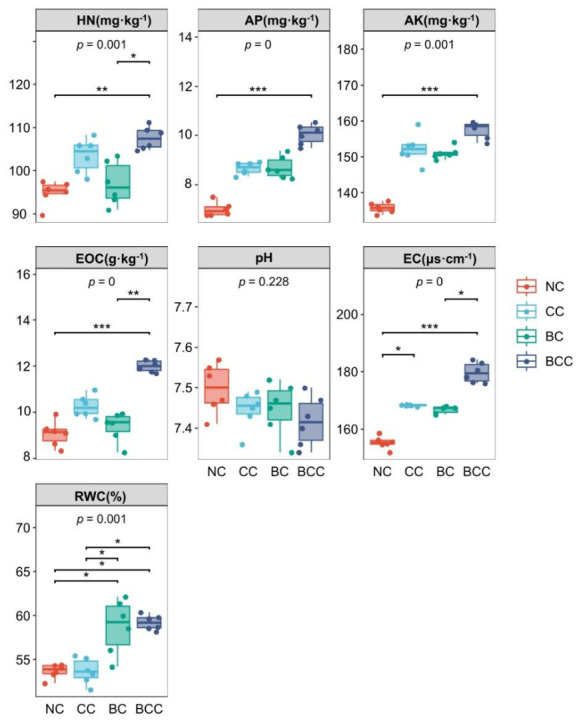
Variations in rhizosphere environmental factors and soil nutrients under different treatments. NC: control group; CC: chemical coating group (without SynCom-SASW01); BC: seed-soaking treatment with SynCom-SASW01 alone; BCC: biological coating treatment combining the SynCom-SASW01 consortium with a chemical coating matrix. HN: hydrolyzable nitrogen content in rhizosphere soil; AP: available phosphorus content in rhizosphere soil; AK: available potassium content in rhizosphere soil; EOC: easily oxidizable organic carbon content in rhizosphere soil; pH: pH value of rhizosphere soil (measured at a soil–water ratio of 1:2.5, *w*/*v*); EC: electrical conductivity of rhizosphere soil (measured at a soil–water ratio of 1:5, *w*/*v*); RWC: soil relative water content, defined as the ratio of actual soil moisture content to field water capacity. Asterisks indicate significant differences: * *p* < 0.05, ** *p* < 0.01, *** *p* < 0.001.

Efficient rhizosphere water utilization is essential for maintaining normal crop physiological metabolism under drought stress. Inoculation with SynCom-SASW01 significantly improved the physical properties of the rhizosphere soil, particularly its water-retention capacity (Figure 3). Under drought conditions, rhizosphere soil RWC in the BCC (biochar-consortium coating) treatment was significantly higher than that in the uninoculated control (NC) group (*p* = 0.001). This increase is likely associated with the EPS-producing capacity of the SynCom identified during preliminary screening, together with its synergistic interaction with the biochar carrier, which may have facilitated the formation of stable “micro-reservoir” around the root system. In addition, the significant elevation in EC suggested an increased concentration of soluble ions in the rhizosphere. This change may contribute to regulation of the soil–plant osmotic potential gradient, thereby enhancing root water uptake capacity.

In addition to water limitation, drought distress is often accompanied by reduced soil nutrient bioavailability. Experimental analysis demonstrated that the concentrations of alkali-hydrolyzable nitrogen (AHN), AP, and AK in the rhizosphere soil were all significantly increased in the BCC treatment (*p* < 0.01). Among these, AP exhibited the greatest increase, rising by approximately 43.17% relative to the NC group, consistent with the strong phosphate-solubilizing capacity of the synthetic community. Meanwhile, AHN content increased from 94.94 mg/kg in the NC group to 107.60 mg/kg in the BCC group. Soil nutrient activation is closely associated with carbon cycling. The BCC treatment significantly increased EOC content by 32.22%, indicating accelerated material turnover and enhanced organic matter transformation within the rhizosphere microenvironment. Despite the significant enhancement of nutrient-related indicators, soil pH remained relatively stable across treatments (*p* = 0.228). This finding suggests that SynCom-SASW01-mediated nutrient optimization was primarily driven by microbial metabolic activities involved in mineral transformation rather than by substantial changes in soil acidity or alkalinity. Collectively, these effects improved the rhizosphere environment under drought stress and provided a stronger material foundation for wheat growth and stress resistance.

### 3.4. Reshaping Effects of SynCom-SASW01 Inoculant on Endosphere and Rhizosphere Microbial Community Structures

**Figure 4:**
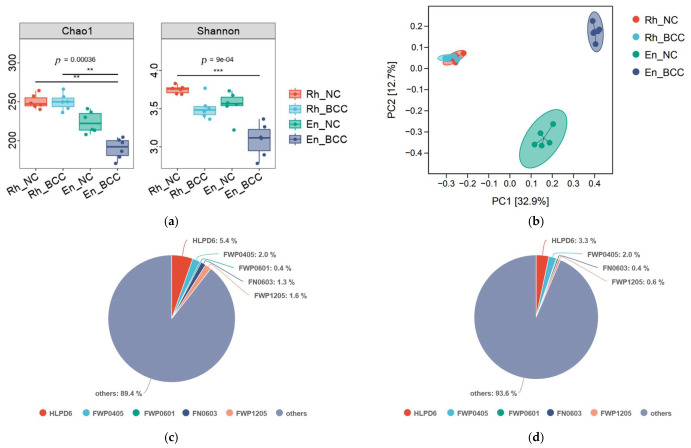

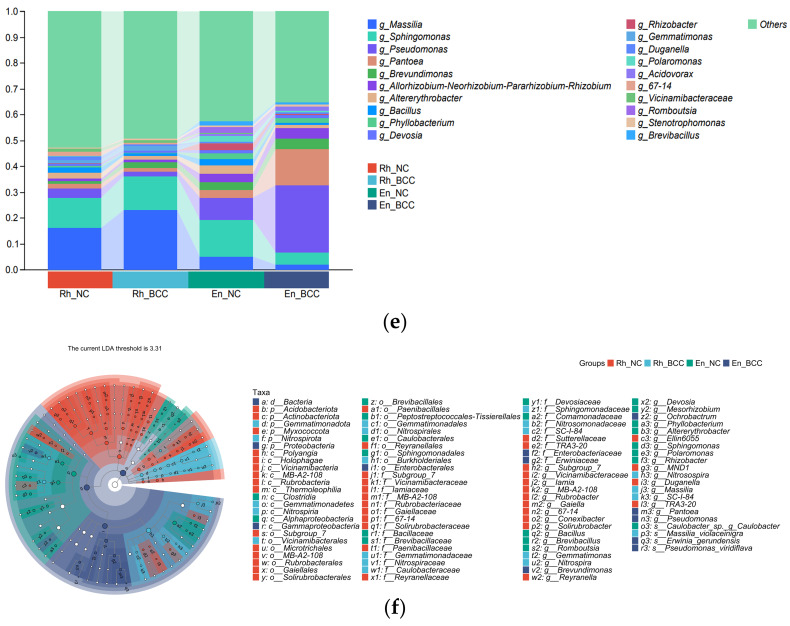
Response patterns of bacterial communities in the endosphere and rhizosphere following the synthetic community (SynCom) treatment. (**a**) Alpha diversity in the endosphere and rhizosphere compartments. (**b**) Beta diversity in the endosphere and rhizosphere compartments. (**c**) Colonization of SynCom-SASW01 strains in the endosphere based on 100% ASV identity. (**d**) Colonization of SynCom-SASW01 strains in the rhizosphere based on 100% ASV identity. (**e**) The bar chart illustrates the composition of the top 20 taxa. (**f**) Identification of core differentially abundant genera based on LEfSe analysis (LDA score > 3). NC: control group; BCC: biological coating treatment combining the SynCom-SASW01 consortium with a chemical coating matrix. En: endosphere zone; Rh: rhizosphere soil zone. Asterisks indicate significant differences: ** *p* < 0.01, *** *p* < 0.001.

The diversity and stability of microbial communities constitute a critical ecological foundation for plants to resist abiotic stresses. In this study, we first evaluated the shifts in the alpha diversity of microbial communities in two microhabitats, namely the wheat rhizosphere (Rh) and endosphere (En), following inoculation with SynCom-SASW01 synthetic microbial consortium. As shown in Figure 4a, under drought stress, inoculation with SynCom-SASW01 significantly increased microbial community diversity in the wheat Rh compared with that in the En, as reflected by both the Chao1 and Shannon indices. This pronounced “niche filtering effect” suggests that wheat roots employ a sophisticated host-selection mechanism to enrich relatively simple yet highly specialized microbial taxa within root tissues. Following inoculation with SynCom-SASW01, alpha diversity in both the Rh and En compartments decreased compared with the control groups (Rh_NC and En_NC). This reduction is likely attributable to a “recruitment effect” of the exogenous consortium, which promoted the homogenization of functional microbial groups during colonization.

Beta diversity analysis further elucidated the successional patterns of community structure across microecological compartments and treatments (Figure 4b). PCoA showed that the first principal coordinate (PC1), explaining 32.9% of the variance, primarily separated samples according to compartment (Rh vs. En). Meanwhile, PC2, explaining 12.7% of the variance, clearly reflected the reshaping effect of the SynCom treatment (BCC). Notably, in the endosphere, En_BCC samples exhibited a clear separation from En_NC samples. These results indicate that SynCom-SASW01 induced a more profound reorganization of the microbiome within root tissues than within rhizosphere soil, suggesting a stronger influence on the internal plant microenvironment.

To identify specific functional community successions driven by the inoculant, we employed Sankey diagrams and LEfSe analysis to identify core differential taxa. The Sankey diagram (Figure 4e) illustrated taxonomic flow from phylum to genus levels. Proteobacteria remained the dominant phylum across all treatments, with *Massilia*, *Sphingomonas*, and *Pseudomonas* constituted the core structural framework of the root microecological community. The LEfSe cladogram (Figure 4f) further revealed that, in the endosphere, the BCC group was significantly enriched with typical PGP taxa, including *Pseudomonas*, Enterobacterales, and *Bacillus*. The directional enrichment of these differential taxa collectively formed a functional collaborative network associated with stress-resistant metabolism. In contrast, the NC group retained a greater abundance of native taxa, such as Rhizobiales and Sphingomonadales.

The ability of exogenous consortia to successfully colonize complex natural communities is a critical indicator for evaluating bioaugmentation efficacy. Using high-throughput sequencing data, we performed abundance-tracking analysis of the core constituent strains of SynCom-SASW01 (Figure 4c,d). The results revealed that all SynCom-SASW01 members achieved successful cross-barrier colonization within the wheat root microecological continuum. Core components such as HLPD6 and FWP0405 reached relative abundances of approximately 3.3–5.4% across different compartments, while strains such as FN0603 maintained stable abundances ranging from 0.4% to 1.3%. This successful colonization, likely facilitated by early-stage niche pre-emption, effectively overcomes the “time-lag effect” associated with host defenses. By establishing a stable ecological barrier, this “intelligent microbiome intervention” provides a stable physiological balance for wheat under drought stress, constituting a core mechanism for enhancing crop resilience.

### 3.5. SynCom-SASW01 Orchestrates Functional Reprogramming of Wheat Root-Associated Microbial Communities Under Drought Stress

**Figure 5:**
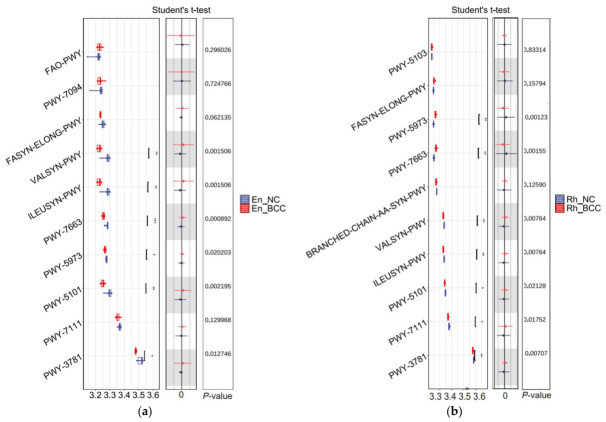
Functional profiles of root-associated bacterial communities. (**a**) Differential functional pathways in the endosphere. (**b**) Differential functional pathways in the rhizosphere. The *y*-axis represents MetaCyc Level 3 pathways. Left *x*-axis: transformed abundance values; Middle: post hoc test results; Right: corresponding *p*-values. NC: control group; BCC: biological coating treatment combining the SynCom-SASW01 consortium with a chemical coating matrix. En: endosphere zone; Rh: rhizosphere soil zone. Statistical significance was evaluated using Student’s *t*-test (* *p* < 0.05, ** *p* < 0.01, *** *p* < 0.001).

Functional potential prediction based on PICRUSt2 and MetaCyc Level 3 pathways (Figure 5) revealed that the microbial functional landscape was predominantly enriched in two major modules: “Biosynthesis” and “Generation of Precursor Metabolites and Energy.” Among the top 20 most abundant metabolic pathways, amino acid biosynthesis, nucleoside and nucleotide biosynthesis, and cofactor and vitamin biosynthesis were dominant. Simultaneously, energy metabolism pathways, including the tricarboxylic acid (TCA) cycle, glycolysis, and fermentation, maintained high relative abundances. Taxonomic contribution analysis demonstrated substantial functional redundancy within the rhizosphere (Rh) compartment, where multiple genera, including *Massilia*, *Sphingomonas* and *Pseudomonas*, collectively contributed to nutrient cycling functions. In contrast, the endosphere (En) compartment exhibited significant functional convergence. Following SynCom-SASW01 inoculation (BCC treatment), the functional contributions of *Pseudomonas* and *Pantoea* within the endosphere were significantly enhanced.

Differential functional analysis of the endosphere (Figure 5a) indicated that SynCom-SASW01 inoculation significantly upregulated several key amino acid biosynthetic pathways. In particular, branched-chain amino acid (BCAA) biosynthesis pathways, including valine biosynthesis (VALSYN-PWY) and isoleucine biosynthesis (ILEUSYN-PWY and PWY-5101), were significant enriched in the En_BCC group (*p* < 0.01). BCAA not only serve as precursors for plant osmolytes but also play a pivotal role in stress signaling transduction. In addition, the enrichment of pathways such as PWY-3781 suggests enhanced carbon utilization efficiency within the endophytic community following inoculation. These shifts in endophytic metabolic potential directly bolstered the host’s internal capacity for ROS scavenging and water balance maintenance.

Significant functional shifts were also observed in the rhizosphere (Rh) compartment (Figure 5b). Compared with the control, the Rh_BCC group exhibited significantly higher abundances of the VALSYN-PWY, ILEUSYN-PWY, and PWY-7111 pathways (*p* < 0.05). Functional remodeling within the rhizosphere was primarily characterized by enhanced capacities for complex organic matter degradation and nutrient activation. The coordinated enrichment of amino acid biosynthetic pathways in both the rhizosphere and endosphere may synergistically support drought resistance and yield maintenance in wheat under drought stress.

### 3.6. Integrative Analysis of the “Microbiome-Physiology-Phenotype” Relationship Mediated by SynCom-SASW01

**Figure 6:**
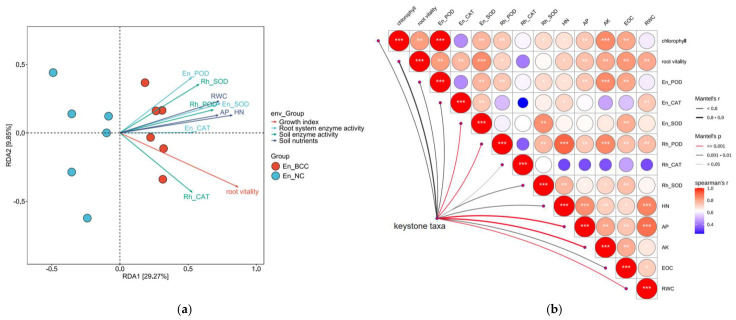
Association analysis among microbial communities, plant physiological traits, and soil environmental variables. (**a**) Redundancy analysis (RDA). (**b**) Network relationships among keystone taxa, multidimensional indicators, Mantel tests, and Spearman’s rank correlations. En: endosphere zone; Rh: rhizosphere soil zone. (* *p* < 0.05, ** *p* < 0.01, *** *p* < 0.001).

To elucidate the relationships between SynCom-SASW01-induced microecological shifts and host drought-tolerance phenotypes, Mantel tests and redundancy analysis (RDA) were conducted to examine associations among microbial communities, wheat physiological traits, and soil environmental factors (Figure 6a). The results indicated that keystone genera enriched by the exogenous inoculant were positively correlated with root vitality, chlorophyll content, and endosphere-associated antioxidant enzyme activities (En_POD, En_SOD, and En_CAT) (*p* ≤ 0.001). These strong correlations profile suggest that keystone genera may play important roles in maintaining host redox homeostasis and photosynthetic capacity. Furthermore, keystone genera were positively associated with rhizosphere soil nutrients, including HN, AP, AK, EOC, and RWC. Notably, Mantel’s r values for HN and AP both exceeded 0.8, indicating that the microbial community directly bolstered plant physiological defense systems by enhancing rhizosphere nutrient mobilization and water retention.

Given that the endosphere compartment exhibited the most pronounced successional shifts following inoculation, RDA was further employed to quantify the contribution of endosphere-associated bacterial community variation to the “phenotype-physiology-nutrient” relationship cascade (Figure 6b). RDA axis 1, explaining 29.29% of the total variance, clearly separated the uninoculated control (En_NC) from the SynCom-SASW01 treatment group (En_BCC). En_NC samples clustered on the left side of axis 1, whereas En_BCC samples shifted rightward along the axis and were closely associated with most positive environmental vectors. Among all environmental variables, root vitality displayed the longest vector and contributed most strongly to axis 1, suggesting that it represents a major phenotypic output deeply regulated by endosphere community shifts. In addition, vectors representing En_POD activity and rhizospheric SOD (Rh_SOD) activity were positively associated with the BCC group, implying that SynCom-SASW01-induced community variation is a core driver for activating the host antioxidant defense system. Regarding nutrient indicators, the vector directions for HN and AP closely aligned with the En_BCC cluster, elucidating why the inoculant treatment significantly improved wheat nutrition under drought stress.

In summary, the exogenous synthetic community efficiently colonized the endosphere and promoted the enrichment of keystone genera such as *Pseudomonas*. These core taxa may contribute to rhizosphere nutrient mobilization, including HN and AP, through their metabolic activities, while simultaneously alleviating drought-induced oxidative stress via modulation of host antioxidant enzyme systems. Collectively, these effects may enhance root vitality and photosynthetic capacity, ultimately contributing to improved wheat productivity under drought stress. This “microecological reshaping–physiological response–phenotypic improvement” cascade provides a mechanistic framework for understanding the synergistic effects of the SynCom-SASW01.

## 4. Discussion

### 4.1. SynCom-SASW01 Stabilizes Rhizosphere Environment Through Enhanced Water-Nutrient Synergy

The rhizosphere serves as the active interface for material exchange between plants and soil, and its physicochemical stability directly influences the severity of drought stress perceived by the plant. This study found that SynCom-SASW01 treatment significantly ameliorated drought-induced water deficits, which may be partly attributable to the EPS secretion capacity of the consortium’s core members. Previous studies have shown that PGPMs can effectively alter the pore structure of rhizosphere soil and form highly hygroscopic biofilms on root surfaces by secreting EPS composed of polysaccharides, proteins, and lipids. These biofilms may function as “micro-reservoirs” that lock in limited moisture and delay root water loss by reducing water loss during soil drying [25]. Alami et al. further verified that EPS-producing strains raised rhizosphere soil water retention by over 20% under drought, consistent with our elevated soil RWC results [26].

SynCom-SASW01 delivered coupled water retention and nutrient activation, providing significant ecological benefits [27,28]. Available phosphorus (AP) rose by 43.17% after inoculation, yet soil pH remained stable (*p* = 0.228), ruling out acid secretion as the primary phosphate-solubilizing pathway. Instead, the consortium likely relied on pH-independent mechanisms including the secretion of phytase and phosphatase. Consistent with the findings of Meharg [29], Rasmann [30], and Zhang et al. [31], synthetic microbial consortia were able to remodel the metabolic profiles of root exudates to mobilize insoluble phosphorus without inducing drastic pH fluctuations. This mild nutrient optimization is especially beneficial for the alkaline or neutral soils of arid regions, as it avoids soil structural degradation associated with acidification. We also observed a significant increase in EOC following SynCom-SASW01 introduction. As a highly sensitive carbon source for soil microbes, the increased EOC abundance reflects both active microbial metabolism and accelerated material cycling within the micro-ecosystem. Fierer’s “carbon source preference” theory suggests that exogenous SynComs can synergistically degrade complex organic carbon, producing small-molecule carbon chains that are easily absorbed by plants, thereby supporting carbon flow within the microbe–soil–plant system under stress [32].

### 4.2. Early Colonization of SynCom-SASW01 Promotes Directional Selection and Functional Succession in the Endosphere

Root tissues serve as selective microbial filters that assemble host-specific endophytic microbiomes. Our data showed significantly lower alpha diversity in the endosphere (En) than the rhizosphere (Rh), with further diversity reduction post-inoculation, supporting the niche filtering theory. Li et al. proposed that drought-stressed plants narrow microbial recruitment via altered root exudate signals to select stress-adaptive functional taxa [33]. SynCom-SASW01 accelerated this screening process, homogenizing endosphere communities toward drought-resistant functions through targeted recruitment.

Stable long-term efficacy depended on the consortium’s capacity to cross soil-rhizosphere–endosphere physical and immune barriers [34]. Sequencing tracking validated persistent colonization of core strains such as HLPD6 inside root tissues. Based on the priority effect theory of Fang et al. [35], microbial assembly order shapes final community function. Seedling-stage inoculation enabled early niche pre-emption, eliminating the lag phase during which plants recruit beneficial microorganisms only after drought damage occurs. This preemptive occupation suppressed pathogen colonization and facilitated the enrichment of key beneficial genera, including *Pseudomonas* and *Bacillus*. LEfSe analysis confirmed their enrichment, matching Berendsen’s “cry-for-help” model, wherein hosts recruit growth-promoting taxa under abiotic stress [36].

SynCom-SASW01 operated as a foundational core consortium to restructure native microflora rather than functioning independently. Members of the genus *Pseudomonas* synthesize ACC deaminase and siderophores to lower ethylene accumulation and delay drought-triggered leaf senescence, which explained the higher chlorophyll concentrations measured in inoculated wheat [37].

### 4.3. Potential Role of the BCAA Metabolic Pathway as a Driver of the “Microbiome-Physiology-Phenotype” Cascade Defense Mechanism

Mantel and RDA correlation analyses revealed that SynCom-SASW01 boosted wheat drought tolerance through a metabolic-physiological-phenotypic cascade, with BCAA biosynthesis as the core regulatory pathway [38]. Valine, leucine and isoleucine serve dual functions as Pro precursors for osmotic adjustment and as alternative respiratory substrates to maintain mitochondrial electron transport when photosynthetic carbon fixation declines under drought [39].

In the present study, the enrichment of the BCAA biosynthetic pathway in the En_BCC group was highly congruent with the two-fold increase in root Pro content. This suggests that SynCom-SASW01 may reinforce the host’s osmoprotective capacity either by enhancing the microbial-derived supply of BCAA or by triggering endogenous BCAA synthesis in the plant via inter-kingdom signaling to strengthen osmotic protection [40,41].

An abundant supply of these metabolic substrates is also believed to contribute to the maintenance of redox homeostasis in wheat under stress [42]. Experimental data revealed that the BCC group exhibited the lowest MDA levels accompanied by markedly increased antioxidant enzyme activities (e.g., SOD and POD) [43,44]. These findings support the hypothesis that SynCom-SASW01 assists the host in establishing a more efficient ROS scavenging system. Consistent with the findings of Abouzari and Fakheri [45], ROS bursts are major triggers of drought-induced cell death; however, microbe-induced systemic resistance (ISR) may place plants in a “primed state,” enabling a rapid physiological response upon stress perception. The synchronized elevation of enzymatic activities observed in both the endosphere and rhizosphere further suggests that this defense effect may involve systemic coordination.

These physiological improvements were ultimately translated into the remodeling of yield components. Increased effective tiller counts (Table 1) stemmed from improved early-stage water use efficiency and nutrient availability, as tiller development highly depends on root vitality and carbon allocation mediated by SynCom-SASW01 [46]. The enhancement of root vitality by SynCom-SASW01 likely ensured a carbon allocation advantage during tiller initiation. Furthermore, the consistent yield improvement observed at both the Hohhot and Hulunbuir sites reflects, to a certain extent, the robust ecological adaptability of this microbiome-mediated stress-resistance system. Overall, the cascade framework of rhizosphere microhabitat reconstruction, host physiological defense activation and improved agronomic phenotypes fully interprets the synergistic drought-resistant effects of SynCom-SASW01. This work provides a practical bioaugmentation strategy to stabilize crop production amid intensifying global drought stress [47].

## 5. Conclusions

Combining indoor pot assays and field trials at two locations (Hohhot and Hulunbuir), this study systematically assessed how SynCom-SASW01 regulated wheat rhizosphere microecology and plant–soil relationships under drought. Grain yields rose markedly at both sites—by 10.42% in Hohhot and 8.77% in Hulunbuir—validating a multi-layer synergistic pathway: microbial community remodeling, soil nutrient activation, and improved plant drought resilience.

SynCom-SASW01 exhibited strong cross-site adaptability. It boosted wheat seedling growth and photosynthesis across semi-arid environments, elevated root vitality and water use efficiency, and steadily raised field yields, proving synthetic consortia worked well in stressful farmland. The inoculant reconstructed rhizosphere habitats. Its secreted EPS boosted soil water retention to form a moisture buffer, while microbial metabolism mobilized mineral nutrients and accelerated EOC cycling. SynCom-SASW01 reshaped the root endosphere more drastically than the rhizosphere. It colonized early via priority effects to bypass host defensive lag responses, filtered niches to enrich beneficial *Pseudomonas* and *Bacillus*, and promoted the establishment of stress-resistant microbial networks. Activated BCAA biosynthesis emerged as a key metabolic pathway contributing to drought tolerance. The consortium stimulated Pro accumulation and antioxidant enzyme activity to clear ROS, relieving drought-triggered lipid peroxidation and cell injury.

## Figures and Tables

**Table 1:** Effect of field inoculation with SynCom-SASW01 on wheat yield at the Hohhot experimental site.

Site	Group	Productive Ear Count (Ear·m^−2^)	Ear Length (cm)	Grains Per Ear (Grain)	1000-Grain Weight (g)	Yield (kg·ha^−1^)
Hohhot	NC	304.17 ± 24.57 d	7.24 ± 0.35 a	23.45 ± 0.99 b	34.83 ± 1.61 a	3568.65 ± 163.73 d
	BCC	333.50 ± 19.80 c	7.74 ± 0.49 a	24.92 ± 1.31 b	34.38 ± 1.67 a	3940.38 ± 241.96 c
Hulunbuir	CC	433.51 ± 22.60 b	7.28 ± 1.43 a	32.76 ± 2.39 a	36.21 ± 1.62 a	4627.50 ± 308.91 b
	BCC	463.81 ± 23.25 a	7.92 ± 0.64 a	31.71 ± 5.70 a	36.13 ± 1.22 a	5021.58 ± 374.73 a

Note: Productive ear count was defined as the number of ears with a length no less than two-thirds of the average ear length and containing at least five filled grains. The 1000-grain weight and grain yield were both measured at a seed moisture content of 13%; NC: control group; BCC: biological coating treatment combining the SynCom-SASW01 consortium with a chemical coating matrix. Different letters indicate significant differences between groups; *p* < 0.05 indicates statistical significance.

## Data Availability

The data presented in this study are openly available in FigShare at https://doi.org/10.6084/m9.figshare.32397822.

## Supplementary Materials

The following supporting information can be downloaded at: https://www.mdpi.com/article/10.3390/microorganisms14071396/s1, Table S1. Basic information of the constituent strains of synthetic community SynCom-SASW01. Table S2. Plant growth-promoting traits of individual strains of SynCom-SASW01. Table S3. Manufacturer and origin information for all materials. Table S4. List of experimental instruments.
