# Supplementary material for: Regulatory Mechanisms of Microbial Consortium Inoculant SynCom-SASW01 in Modulating Rhizosphere–Endophytic Interactions and Enhancing Drought Resistance in Wheat
Source: Microorganisms. 2026 Jun 24;14(7):1396. doi: 10.3390/microorganisms14071396 (PMC13414153; doi:10.3390/microorganisms14071396)
Supplement: Supplementary file 1 [file microorganisms-14-01396-s001.zip › microorganisms-4368566-supplementary.pdf]

Regulatory Mechanisms of Microbial Consortium Inoculant SynCom-SASW01 in Modulating Rhizosphere–Endophytic Interactions and Enhancing Drought Resistance in Wheat

Table S1. Basic information of the constituent strains of synthetic community SynCom-SASW01.

Strain ID	Coordinates	Host Plant	Isolation Site	Closest Species	GenBank Accession No.
FN0603	E:110.48972°, N:40.34138°	*Triticum aestivum* L.	Root endospheric	*Enterobacter hormaechei*	PQ226002
FWP0601	E:102.68764°, N:39.40429	*Zygophyllum xanthoxylum* (Bunge) Maxim.	Root endospheric	*Enterobacter cloacae*	PQ226000
FWP0405	E:105.62500°, N:38.67572	*Salicornia europaea* L.	Root endospheric	*Pseudomonas putida*	PQ225999
FWP1205	E:105.62500°, N:38.67572	*Salicornia europaea* L.	Root endospheric	*Enterobacter ludwigii*	PQ226001
HLPD6	E:104.47603°, N:37.48512	*Haloxylon ammodendron* (C. A. Mey.) Bunge	Root endospheric	*Stenotrophomonas maltophilia*	PQ226003

Table S2. Plant growth-promoting traits of individual strains of SynCom-SASW01.

Plant Growth-Promoting Traits / Strain ID	FN0603	FWP0601	FWP1205	FWP0405	HLPD6
Nitrogen fixation capacity (nmol C_2H_4 /h/mg protein)	47.66	32.26	39.93	33.15	24.22
Phosphate solubilization capacity (mg/L)	29.99	-	27.97	33.83	22.43
Siderophore production (%)	56.55	67.88	45.33	26.89	9.58
ACC deaminase activity (ng/mg protein)	4.47	4.69	4.63	3.29	4.25
IAA production (mg/L)	21.86	18.97	17.17	2.15	0.64
Biofilm formation ($OD_{590\ nm}$)	0.26	0.26	0.26	0.56	0.55

EPS production (μg/mL)	206.27	194.08	210.8	226.67	145.78
Salt tolerance (NaCl %)	0~10	0~10	0~8	0~10	0~7
Tolerable pH range	6.5~10	6.5~10	6.5~10.5	5.5~9	6~9
Bacterial protein content (μg/mL)	55.46	58.02	46.37	52.72	51.39

Note: "—" indicates no detectable activity. The bacterial protein content was measured when the OD_{600} nm of the bacterial suspension reached 1.0.

Table S3. Manufacturer and origin information for all materials.

Reagent	Manufacturer	Country of Origin
Biochar (Particle size 45 μm)	Tanluzhe Co., Ltd.	China
Trehalose	Annuo Food Chemical Co., Ltd.	China
Maltodextrin	Annuo Food Chemical Co., Ltd.	China
Phosphorus-potassium mineral powder (Total P 110 g/kg, Total K 10 g/kg)	Hongsheng Chemical Technology Co., Ltd.	China
Molasses	Annuo Food Chemical Co., Ltd.	China
Fulvic acid	Guangdong Puhui Chemical Technology Co., Ltd.	China
Potassium silicate	Hongsheng Chemical Technology Co., Ltd.	China
Hoagland's nutrient solution	Xiqingchun Agricultural Technology Co., Ltd.	China

Note: All reagents used in this study are industrial-grade products.

Table S4. List of experimental instruments.

Instrument Name and Model	Manufacturer	Country of Origin
FA-2004B Electronic Analytical Balance	Shanghai Jinghai Instrument Co., Ltd.	China
BD-307HNE Laboratory Freezer	Qingdao Aucma Co., Ltd.	China
DW-HL528S Ultra-low Temperature Freezer	Meiling Cryogenic Technology Co., Ltd.	China
SKE-5S Ultrasonic Cleaner	Ningbo Yinzhou Shuoli Instrument Co., Ltd.	China
VG-3-S25 Vortex Mixer	IKA Works Guangzhou	China
TissueLyser II Tissue Grinder	Qiagen	China
Hitachi CF16R Rx II High-speed Refrigerated Centrifuge	Techcomp Scientific Co., Ltd.	China
SX-500 Automatic High-pressure Autoclave	TDMY KOGYO,	Japan

SFZ-82 Constant Temperature Shaker	Shenzhen Guohua Instrument Factory	China
Bat40-2LGF Electric Thermostatic Blast Drying Oven	Wuhan Boante Instrument Technology Co., Ltd.	China
ABI GeneAmp® 9700 PCR System	Thermo Fisher Scientific	America
INFINITE 200pro Microplate Reader	Tecan	Switzerland
SPAD-502plus Portable Chlorophyll Meter	Konica Minolta	Japan
HI-2221 pH Meter	Hanna Instruments	Italy
DDBJ-350 Portable Conductivity Meter	Shanghai Precision Scientific Instrument Co., Ltd.	China
2.5/10/50/100/200/1000 μL Pipette Set	Eppendorf	Germany
BSC-1300 II A2 Biosafety Cabinet	Suzhou Antai Air Technology Co., Ltd.	China
HWS-26 Electric Thermostatic Water Bath	Shanghai Hengke Scientific Instrument Co., Ltd.	China
